# Aggressive vertebral hemangioma: a post-bioptic finding, the gas web sign—report of two cases

**DOI:** 10.1259/bjrcr.20190091

**Published:** 2020-09-29

**Authors:** Young-wouk Kim, Lokmane Taihi, Flore Viry, Philippe Bossard, Marc Polivka, Valérie Bousson

**Affiliations:** 1 Service d’Imagerie Ostéo-Articulaire, Hôpital Lariboisière, AP-HP, 2 rue Ambroise Paré, 75010 Paris, France; 2 Université Paris Diderot, CNRS UMR 7052, 75010 Paris, France; 3 Service d’Anatomopathologie, Hôpital Lariboisière, AP-HP, 2 rue Ambroise Paré, 75010 Paris, France

## Abstract

Vertebral hemangiomas are relatively frequent among tumors of the spine. Most of them are asymptomatic and the diagnosis is usually made based solely on imaging. However, although rare, some hemangiomas with atypical imaging features (aggressive hemangiomas) can pose a diagnostic challenge. Clinically, these patients present with neurological symptoms. In imaging, aggressive hemangiomas appear as lesions with significant osseous expansion or extraosseous extension, mimicking the appearance of other tumors, such as metastasis or plasmacytoma. In such cases, a biopsy is often required to obtain a histopathological diagnosis in order to rule out the differential diagnoses mentioned above. We report on two cases of aggressive hemangiomas whose diagnosis remained uncertain until the pathology analysis. On CT-scan control immediately after biopsy, we have been surprised to observe the formation of gas bubbles inside the biopsied lesion, spreading over almost the whole vertebra. This gas web sign may support its liquid-filled spaces composition and its benign nature. Our goal was to highlight this finding and its usefulness.

## Introduction

Vertebral hemangiomas are relatively frequent among tumors of the spine. Most of them are asymptomatic and the diagnosis is usually made based solely on imaging. However, some hemangiomas with extensive osteolysis, cortical expansion or associated with a soft tissue mass (aggressive hemangiomas) can pose a diagnostic challenge, mimicking the appearance of other tumors, such as metastasis or plasmacytoma, therefore requiring a histopathological diagnosis. We report on two cases for which the diagnosis of vertebral hemangioma remained uncertain until the pathology analysis. On CT-scan control immediately after biopsy, we have been surprised to observe the formation of gas bubbles inside the biopsied lesion, supporting its liquid-filled spaces composition and its benign nature. Our goal was to highlight this finding and its usefulness.

## Case report

### Case 1

**Figure 1.**
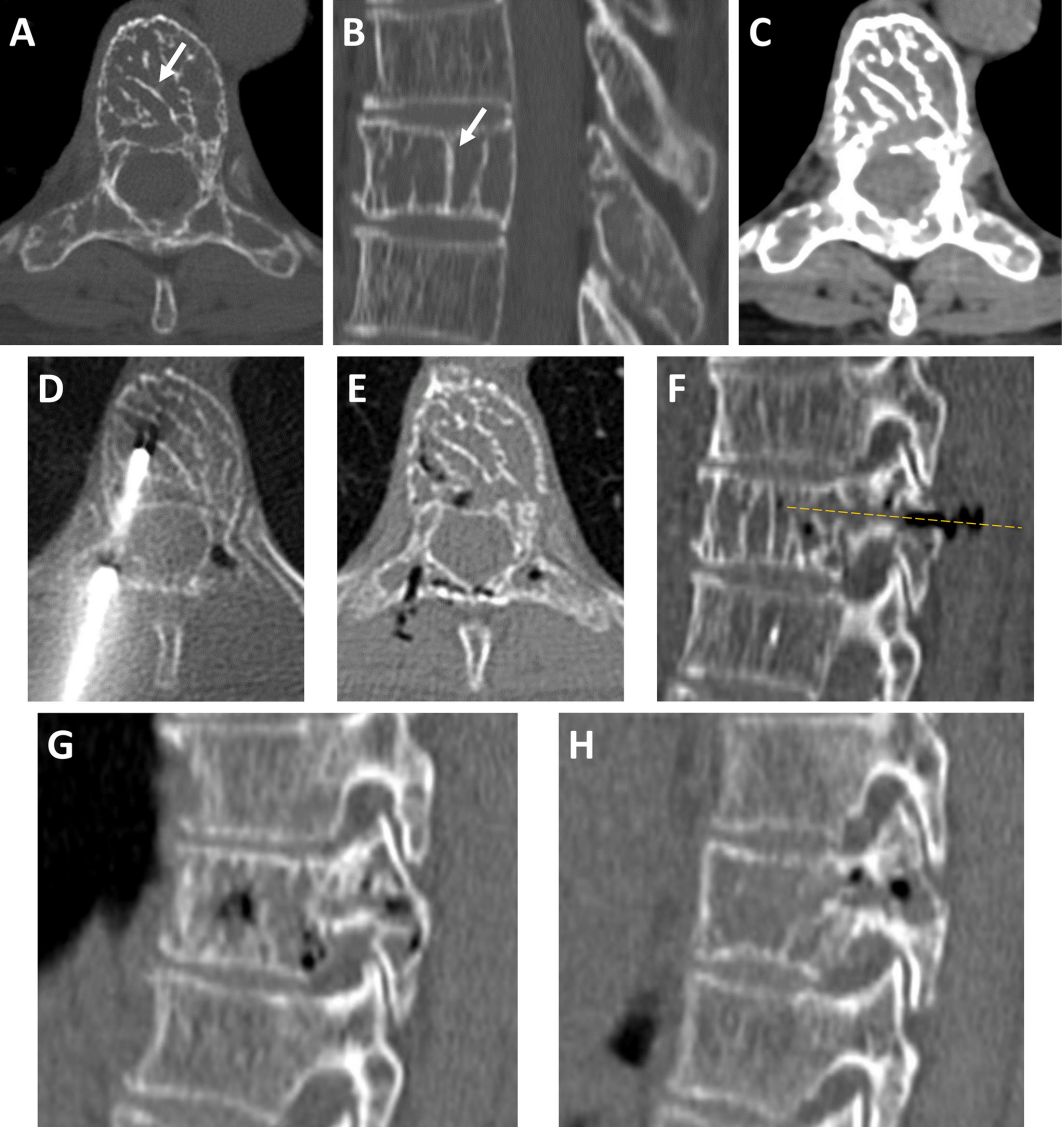
CT-scan of an osteolytic lesion of the T8 vertebra, composed of thickened vertical trabeculae *[arrows in axial slice (A) with sagittal reconstruction (B) in bone window*]. The lesion involved the vertebral body and the posterior arch, with some degree of hyperostosis of the whole vertebra [enlargement of the anteroposterior diameter in *(B*)]. There was only mild paravertebral soft tissue extension (*C: axial slice in soft tissue window*). After CT-guided biopsy through the right pedicle (*D*), gas bubbles have formed along the biopsy path and remotely inside the lesion (*E*). Sagittal reconstruction better shows the biopsy path through the pedicle (*dotted yellow line*), partially filled with gas bubbles (*F*). Note the presence of gas bubbles in both pedicles after biopsy (*G: right pedicle in sagittal slice; H: left pedicle in sagittal slice*).

**Figure 2.**
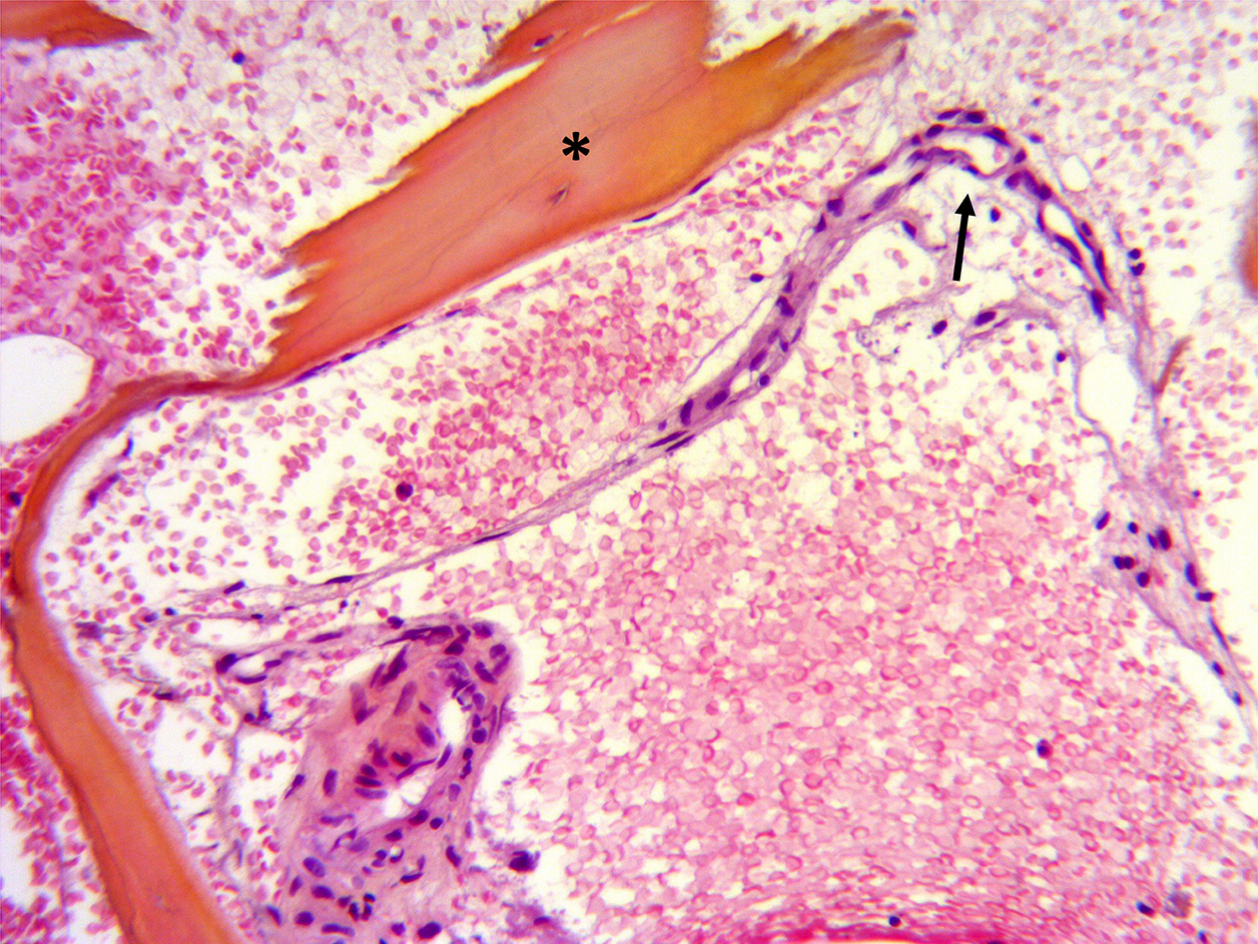
Pathologic result of the lesion of the T8 vertebra of the first patient (Figure 1),showing thickened trabeculae (*) and thin walled capillary vessels (*arrow) (hematoxylin eosin safran coloration; magnification ×200*).

A 60-year-old female presented with chronic mechanical cervicobrachial neuralgia and paresthesia of the three last fingers of her right hand. There was no trigger factor. The pain was intermittent and moderate, but it was worsening over time in the past few weeks. She had no known medical history and took no drug therapy. The physical examination was unremarkable, in particular, she had no motor deficit. MRI showed no disc herniation but a lesion of the T8 vertebra that was further characterized by a CT-scan. It was an osteolytic lesion of the vertebral body with right and left pedicle and lamina extensions, containing thickened vertical trabeculae (Figure 1; A–B﻿). There was some degree of expansion of the whole vertebra, assessed by the enlargement of the anteroposterior diameter (Figure 1; B). The paravertebral soft tissue extension was mild (Figure 1; C). There was no other vertebral lesion. Differential diagnoses considered at this moment were aggressive hemangioma, plasmacytoma and, to a lesser extent, Paget’s disease. Blood test showed no abnormality except an IgM κ monoclonal gammapathy. A biopsy was performed under CT-guidance and samples were addressed to the pathology department. A systematic CT-scan control immediately after the biopsy showed the formation of gas bubbles along the path of the biopsy, but also remotely inside the vertebral body (Figure 1; E–H). Pathology analysis revealed thickened trabeculae and medullary spaces filled with blood and thin-walled capillary vessels (Figure 2). There was no evidence of tumoral proliferation. The diagnosis of hemangioma was therefore retained.

### Case 2

**Figure 3.**
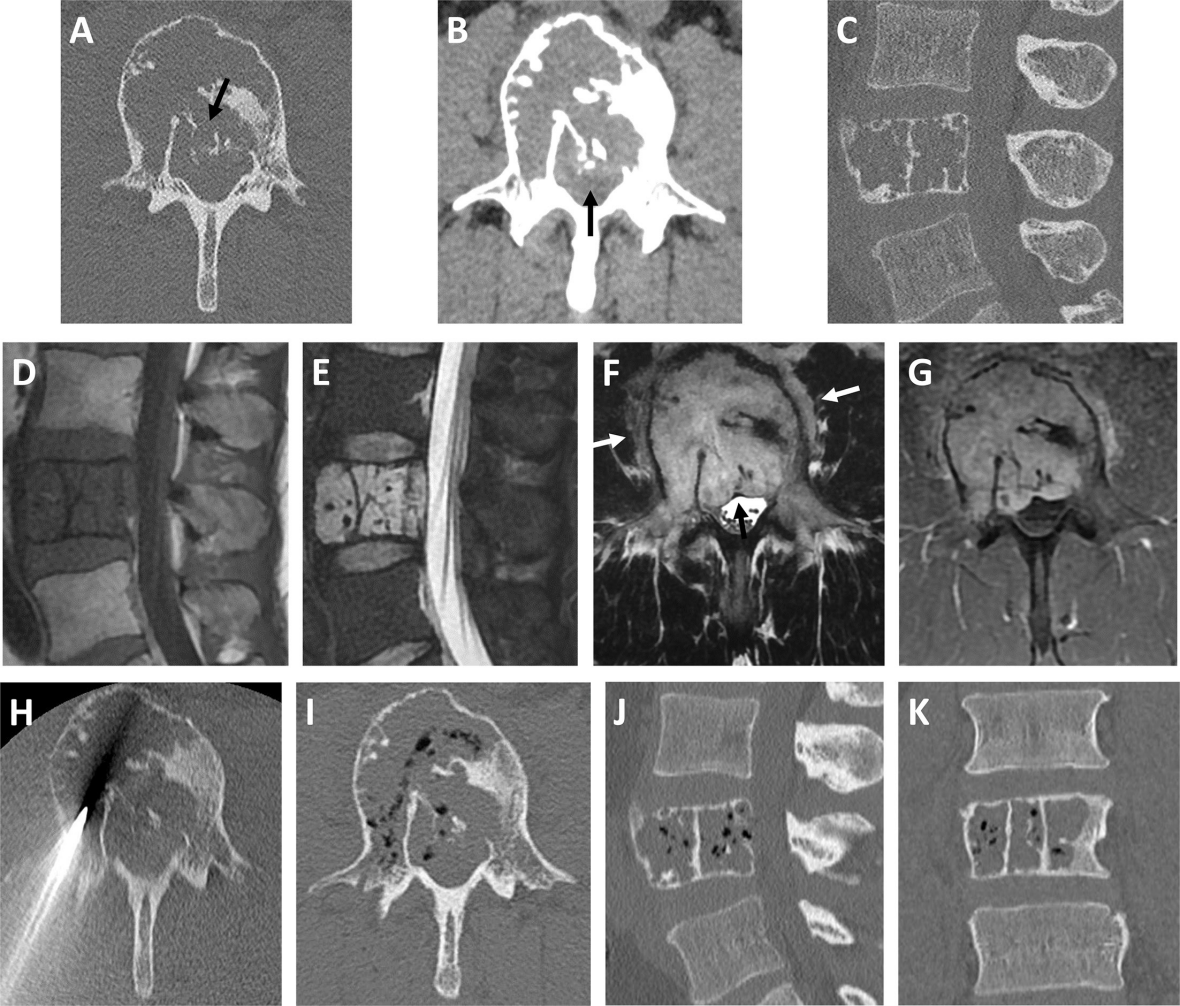
Expansile osteolytic lesion of the L4 vertebra, involving mostly the vertebral body with rupture of cortices in places (*arrow in A: axial slice in bone window CT-scan*)with posterior protrusion inside the spinal canal (*arrow in B: axial slice in soft tissue window CT-scan*). The lesion was composed of thickened vertical trabeculae (*C: sagittal reconstruction in bone window*). MRI showed a low signal on *T*_1_ weighted images (*D: sagittal slice*)and high signal on STIR-weighted images (*E: sagittal slice*), with invasion of the surrounding soft tissue and posterior protrusion inside the spinal canal (*arrows in F: axial T_2_ weighted slice*). The lesion was strongly and homogeneously Gadolinium-enhanced *(G: axial T_1_ weighted contrast-enhanced slice)*. After CT-guided biopsy *(H)*, gas bubbles have formed along the biopsy path and inside the lesion, notably in its epidural component *(I)*. The diffusion of gas bubbles inside the vertebral body is also shown in sagittal *(J)* and coronal *(K)* reconstructions. STIR, shorttau inversion recovery.

**Figure 4.**
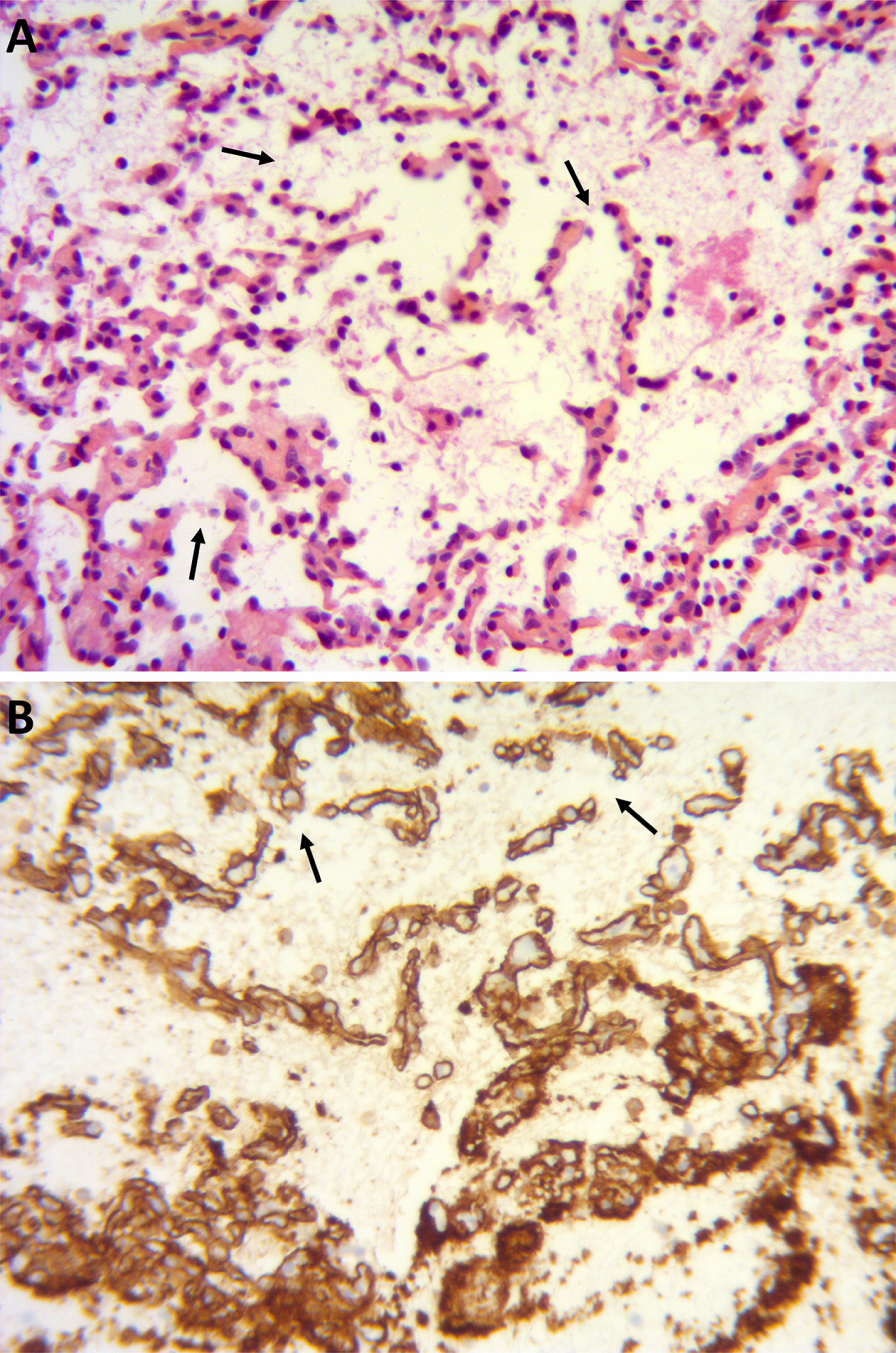
Pathologic result of the lesion of the L4 vertebra of the second patient (Figure 3), showing some thickened trabeculae but mostly thin-walled dilated capillary vessels *(A: hematoxylin eosin safran coloration; magnification ×200)*. Anti-CD31 staining was positive *(B)*, confirming the vascular origin of the lesion. Note the largely porous borders of the capillary vessels *(arrows in A, B)*.

A 43-year-old male presented with moderate mechanical lower back pain and right anterior thigh pain for 3 months, onset after a jog. There was no radiculalgia, pain, numbness nor weakness of the leg. He had no significant past medical history and took no drug therapy. The physical examination was unremarkable; there was no sensory or motor loss. Blood test was normal. CT-scan showed an expansile osteolytic lesion of the L4 vertebra, involving mostly the vertebral body with rupture of cortices in some places (Figure 3; A–C). A MRI was then performed and showed a low signal on *T*_1_ weighted images (Figure 3; D) and high signal on short tau inversion recovery (STIR)-weighted images (Figure 3; E), with invasion of the surrounding soft tissue and posterior protrusion inside the spinal canal (Figure 3; F). The lesion was strongly and homogeneously Gadolinium-enhanced (Figure 3; G). Differential diagnoses considered at this moment were aggressive hemangioma and plasmacytoma. Due to these imaging features, a biopsy has been performed under CT-guidance. On control CT-scan, we also observed the formation of gas bubbles along the path of the biopsy, but also remotely inside the vertebral body (Figure 3; I–K). Pathology analysis including an anti-CD31 staining concluded to a hemangioma (Figure 4).

## Discussion

Vertebral hemangioma is relatively frequent and is the most common primary tumor of the spine.^1^ It accounts for approximately 10% at general autopsy.^2^ On pathology, they are composed of thin-walled, blood-filled vessels lined with a single layer of flat endothelial cells. The vessels permeate the bone marrow and surround pre-existing trabeculae. Secondary reactive phenomena then occur, such as fibrous and/or adipose involution of bone marrow and remodeling of bone trabeculae.^3^ Histologically, hemangiomas are divided into capillary, cavernous, arteriovenous and venous types. However, these lesions are often not histologically pure, showing variable histology.^4^

Hemangiomas are located in spine in 70% of the cases (notably in thoracic spine) and in skull in 10–20% of the cases. They have distinctive histological findings depending on the amount of the fat, interstitial edema and blood vessels. The majority of vertebral hemangiomas are asymptomatic and have common characteristic imaging features. However, approximately 1% of vertebral hemangiomas produce neurological symptoms.^3,5^ The neurological symptoms are due to cord or nerve compression, which results from a hypertrophy or fracture of vertebral body, an extension of hemangioma into epidural space, a hemorrhage or anomalous vessels draining or feeding the lesion.^5^

Vertebral hemangiomas can demonstrate various imaging patterns. The variations of signal intensity in the MR images correlates with the histological picture, as the composition of the lesion varies between fatty tissue, vasculature and interstitial edema.^6^ For example, the presence of fatty component is indicative of inactive form of vertebral hemangioma, whereas lesions with low signal intensity on MRI indicate more active vascular type with potential to compress the spinal cord.^7^ Typical vertebral hemangioma appears as a “polka dotted” vertebra at CT-scan due to thickened vertebral trabeculae, in reference to a clothing pattern consisting of equally sized and spaced filled circles.^8^ When seen on sagittal and coronal images, hemangiomas appear as vertically oriented thickened trabeculae, known as the “corduroy sign”. On MRI, vertebral hemangiomas are hyperintense on both *T*_1_- and *T*_2_ weighted imaging, mildly hyperintense on STIR sequence. Thickened trabeculae appear as low signal areas on both *T*_1_- and *T*_2_ weighted imaging.^8^ These lesions show variable enhancement after contrast administration. Some vertebral hemangiomas differ from typical hemangiomas in the form of increased vasculature with reduced or absent fat content, mostly assessed by MRI. They appear as iso- to hypointense on *T*_1_ weighted imaging, and hyperintense on *T*_2_ weighted and STIR imaging. Aggressive hemangiomas refer to lesions with significant osseous expansion or extraosseous extension. Features such as involvement of the entire vertebral body, extension to the neural arch, expanded cortex with indistinct margins and soft-tissue mass have been described in aggressive hemangiomas.^9^ On MRI, they appear hypointense on *T*_1_ weighted images and hyperintense on *T*_2_ weighted and STIR images. Diffusion-weighted imaging has been used to differentiate hemangioma and metastasis in difficult cases, and found it to be a useful tool in the diagnosis, characterization and differentiation of both.^10^ At angiography, aggressive vertebral hemangiomas show characteristic dilatation of arterioles of vertebral body, multiple blood pools in the capillary phase and intense opacification throughout the vertebral body. Vertebral hemangiomas are typically ametabolic on ^18^FDG-PET scanning with no uptake on bone scan. However, rarely they may have atypical imaging features and appear hypermetabolic.^11^

Although typical hemangioma can be easily diagnosed based solely on imaging, some aggressive hemangiomas can pose a challenge. Indeed, hypervascular metastases, plasmacytomas and lymphomas can share the same expansile osteolytic appearance on imaging. Metastases to the spine are commonly multiple and involve the pedicle and posterior vertebral elements. There is also a history of primary tumor most of the time. Plasmacytomas can show marked erosion, expansion and destruction of the bone cortex. Lymphomas usually appear as lesions with associated epidural, paraspinal soft tissue masses and homogeneous enhancement on contrasted MRI. In some cases, Paget's disease of the spine can have a similar appearance but is usually distinguished by expansion of the vertebral body with peripheral cortical thickening demonstrated on CT-scan.

In such cases, where diagnosis remains uncertain after imaging, a pathological diagnosis is required, generally by a CT-guided biopsy, considering that the management differs between hemangioma and the other differential diagnoses mentioned above. Indeed, as majority of vertebral hemangiomas detected are asymptomatic, no specific treatment is required. For the other cases, the treatment options are many, including surgical decompression, percutaneous vertebroplasty with polymethyl methacrylate, intralesional injection of ethanol, intra-arterial embolization and radiotherapy, even though this latter practice remains controversial.^3,12^

We presented the case of two patients with aggressive vertebral hemangioma whose diagnosis was uncertain on imaging but confirmed at histopathology, in order to describe a possible new post-bioptic finding. We describe the formation of gas bubbles inside the biopsied vertebra, notably at distance from the biopsy path. We can only speculate on the origin of the gas inside these lesions. The gas might have penetrated from the outside through the trocar and diffused through the entire lesion between trabeculae. The capacity of the lesion to let enter and dispatch gas bubbles supports the idea that the lesion is mainly composed of liquid-filled spaces. Indeed, only a liquid lesion could let the gas pass through, contrary to solid tumor, such as plasmacytoma. Besides, the presence of vessels organized in cavities interconnected via porous walls seen in pathology could explain the ability of the gas to diffuse through the lesion. We have also noted that the gas was arranged in a linear fashion, as though it was confined to tubular structures (such as vascular channels seen in pathology). Due to these imaging features, and in particular the capacity of the gas to form and to spread over almost the whole vertebra, we find the term of “gas web sign” the most appropriate to depict this finding.

The presence of gas inside a vascular lesion has been reported in lymphangiomatosis of bone.^13^ The authors assumed that the fluid in these lesions may have resorbed spontaneously, creating negative pressure and allowing nitrogen gas from the blood to accumulate in them. Nevertheless, all their patients underwent head and neck surgery before pneumatosis was detected. Similarly, the presence of gas bubbles in vertebrae was described in osteoporotic fracture as the “intravertebral vacuum phenomenon,” and constitutes an argument for benign vertebral fractures as opposed to malignant fractures.^14–16^ During the biopsy procedure, our patients were placed prone on the CT table. This positioning can be assimilated to a spine hyperextension, point that can be placed in parallel with the vacuum phenomenon appearing during extension of the spine.^14,15^

In clinical practice, this sign can be interesting for two reasons. Firstly, because biopsy of aggressive hemangioma may present a risk of bleeding, with occurrence or worsening of neurological symptoms. It is therefore potentially hazardous to obtain several samples. Observing gas bubbles at the first track prevents from performing additional samples. Secondly, because samples are usually quite small or quality being compromised in case of hemorrhage, negative results at pathology are not rare. The formation of gas bubbles inside the lesion after biopsy, freely diffusing through the whole lesion, could become an additional argument in favor of the diagnosis of hemangioma. However, these results are preliminary and may need further research to establish the usefulness of this diagnostic sign.

## Conclusion

We presented two cases of aggressive vertebral hemangiomas, whose diagnosis remained uncertain at imaging, thus requiring pathological confirmation. We have noted a post-bioptic finding consisting in the formation of gas bubbles freely diffusing through the whole lesion after the biopsy, in favor of its liquid-filled spaces composition and its benign nature. This gas web sign may be of additional value in non-conclusive cases favoring aggressive vertebral hemangioma, but there will be further studies needed to assess its value and accuracy.

## Learning points

- Aggressive vertebral hemangioma can present with significant osseous expansion or extraosseous extension on imaging, therefore mimicking other tumors, such as metastases, plasmacytomas or lymphomas.
- In such cases, a pathological diagnosis by biopsy is required.
- The formation of gas bubbles inside the biopsied lesion and along the biopsy path (“gas web sign”) may be of additional value in non-conclusive cases favoring aggressive vertebral hemangioma. However, these results are preliminary and may need further research to establish the usefulness of this sign.
